# SpyTag/SpyCatcher-mediated protein ubiquitination to investigate 20S and 26S proteasomal degradation

**DOI:** 10.1039/d5sc05440k

**Published:** 2025-09-10

**Authors:** Julia Kriegesmann, Shahar Levi, Mahdi Hasan, Eman Nassar, Michael Glickman, Ashraf Brik

**Affiliations:** a Schulich Faculty of Chemistry, Technion – Israel Institute of Technology Haifa Israel abrik@technion.ac.il; b Faculty of Biology, Technion – Israel Institute of Technology Haifa Israel

## Abstract

Ubiquitination significantly influences human health and disease because it plays an essential role in many cellular signaling pathways. To investigate the effects of different types of ubiquitination, various strategies based on synthesis, semisynthesis, or expression have been developed for protein ubiquitination. Here, we introduce a new method for protein ubiquitination using the SpyTag/SpyCatcher system. By combining protein expression with chemical synthesis, we created enhanced green fluorescent protein (eGFP) with ubiquitin chains consisting of 1–4 units linked through Lys48. This allowed us to study how different ubiquitin chains affect proteasomal degradation by the 26S and 20S proteasomes. While the 26S proteasome only trimmed the ubiquitin chain, the 20S proteasome degraded the different ubiquitin variants, highlighting the flexibility of the 20S proteasome in degrading complex ubiquitinated proteins.

## Abstract 2

By combining protein expression with chemical synthesis, we created enhanced green fluorescent protein with ubiquitin chains consisting of 1–4 units linked through K48 to study how they affect proteasomal degradation by the 26S and 20S proteasomes.

## Graphical abstract

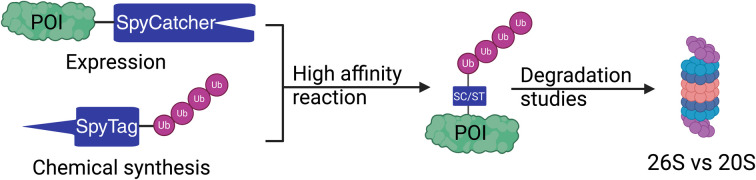

## Introduction

Ubiquitination, which involves attaching ubiquitin (Ub) monomers or Ub chains to protein substrates, is one of the most significant post-translational modifications (PTMs) influencing a wide range of cellular processes, including proteasomal protein degradation, intracellular trafficking, and DNA damage response.^1,2^ The transfer of Ub or Ub units to the target protein is catalyzed by three enzymes: E1 Ub-activating enzymes, E2 Ub-conjugating enzymes, and E3 Ub-protein ligases. In these conjugates, the C-terminal carboxy group of Ub links to the ε-amine of a Lys residue or the N-terminal amine,^3^ and, to a lesser extent, to the side chain of Ser/Thr/Cys^4^ in the protein substrate. Since all seven Lys residues within Ub (K6, K11, K27, K29, K33, K48, K63) and Met1 can be ubiquitinated, forming different types of polymeric chains, ubiquitination is involved in a wide variety of cellular processes.^5^ Ubiquitination is reversed by a family of enzymes known as deubiquitinases (DUBs), which remove Ub or Ub chains from proteins.^6,7^ Since ubiquitination and deubiquitination are involved in many cellular pathways, they significantly influence human health and disease. Therefore, understanding this complex PTM is crucial for basic research and the development of novel therapeutics for various diseases.

Lys48-linked ubiquitination marks target proteins for degradation by the ubiquitin–proteasome system, which is the primary proteolytic pathway.^8^ The type and length of the Ub chain influence the degradation of a protein. However, various hypotheses exist regarding the optimal ubiquitination pattern for effective protein degradation. While it has been proposed that tetra-Ub is the minimal length for proteasomal degradation,^9^ other studies have shown that multiple di-Ub or even single mono-Ub can be a sufficient degradation signal.^10–13^ These different findings can be explained by the fact that the proteasome is often found as a mixture of 30S, 26S, and 20S complexes, which makes it challenging to dissect the specific role of each part.

The 26S proteasome consists of a 19S regulatory particle, which recognizes the Ub signal and unfolds the target protein, and a 20S core particle, which hydrolyzes the unfolded protein into short peptides. Although the 20S is a part of the 26S proteasome, it is abundant as a free complex in many cell types.^14^ The cellular ratio between the 20S and the 26S proteasome may change as part of an adaptive response to meet cellular needs.^15^ While the ubiquitin on a protein is essential for its binding and proteolysis by the 26S proteasome, the 20S proteasome still has no identified Ub receptors and rapidly degrades proteins in a Ub-independent manner.^16^

**Fig. 1:**
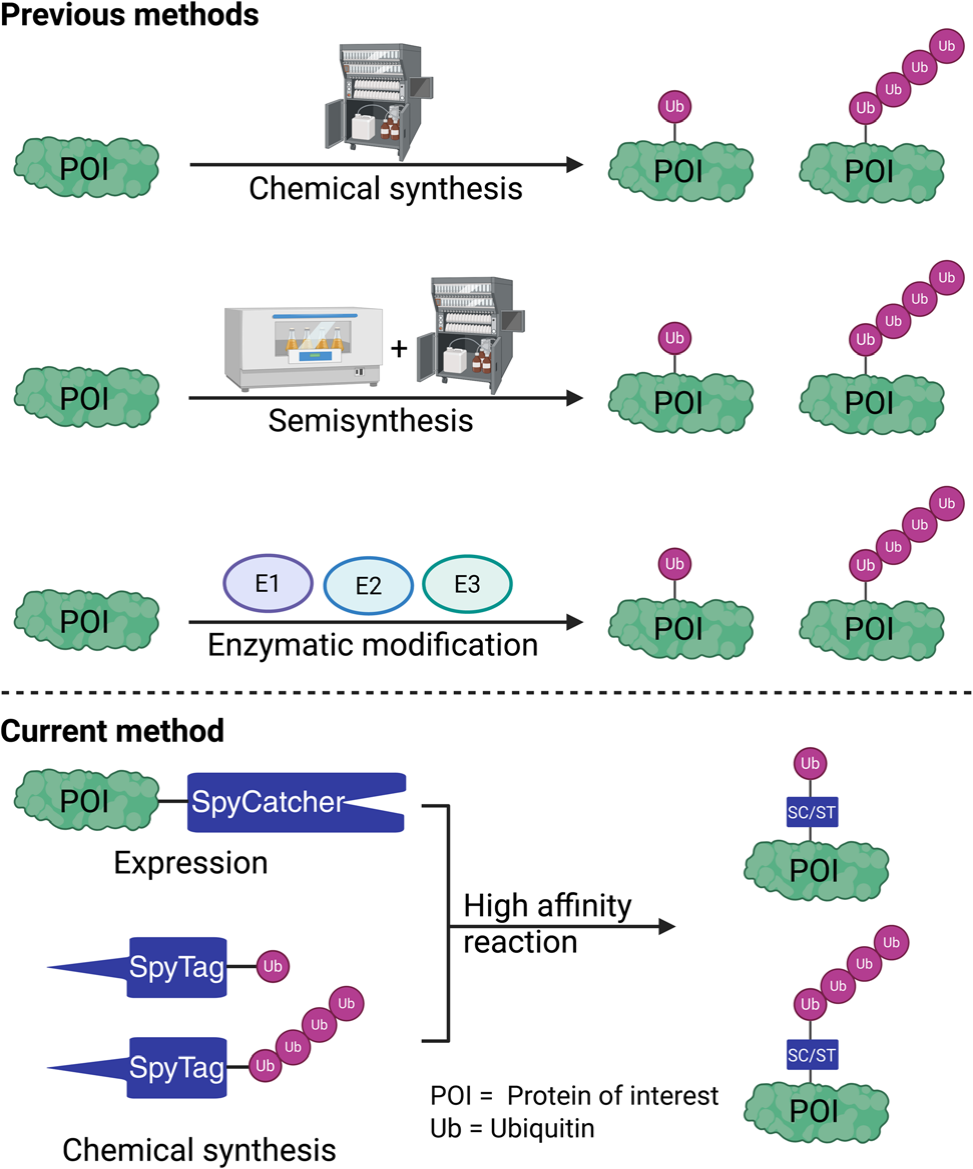
The previously reported synthetic or semi-synthetic strategies for preparing ubiquitinated proteins and our current method, which utilizes the SpyTag/SpyCatcher system.

To study the differences between the 20S and 26S proteasomal degradation in terms of their substrate selection and to thoroughly understand the influence of different ubiquitin chains on proteasomal degradation, homogeneously modified ubiquitinated proteins in workable quantities must be prepared efficiently and cost-effectively. Generating homogeneously ubiquitinated proteins through enzymatic methods poses challenges, as most E3 ligases are promiscuous and can lead to the ubiquitination of target proteins on multiple lysine residues, resulting in mono- and polyubiquitination.^17^ Therefore, several strategies based on protein expression, chemical synthesis, and semisynthesis have been developed over the last two decades (Fig. 1).^18^

Chemical strategies for preparing ubiquitinated proteins primarily rely on chemical ligation of unprotected peptides.^18^ We have, for example, installed δ-mercaptolysine into different proteins for their mono- and polyubiquitination.^16,19–21^ Other strategies are based on expressed protein ligation (EPL),^22^ auxiliary site-specific ubiquitination^23^ or on linking the Ub and the respective target protein by triazole,^24^ dibromoacetone,^25^ disulfide bonds,^26,27^ oxime linkages^28^ or thioether.^29^

Although various methods for generating ubiquitinated proteins with either native or unnatural linkages between the ubiquitination and the substrate have been developed, they predominantly yield monoubiquitinated proteins.^18^ Strategies for preparing protein substrates with longer chains, such as tetraubiquitinated proteins, are still limited. Current synthetic methods are time-consuming and expensive, underscoring the need to expand the available techniques by combining, for example, chemical and enzymatic approaches.^30,31^

Here, we present a new strategy for the preparation of ubiquitinated proteins based on the SpyTag/SpyCatcher system, which integrates chemical synthesis, protein expression, and the reactivity of the SpyTag/SpyCatcher system to bridge the different building blocks into final Ub conjugates. By utilizing the new system, we generated a set of mono-, di-, tri-, and tetra-ubiquitinated proteins to examine their behavior with the 20S and 26S proteasomes.

## Results and discussion

We recently employed total chemical synthesis to generate a panel of homogenously ubiquitinated Cyclin B1-NT variants, enabling a direct comparison of 20S and 26S proteasomes in terms of substrate recognition and peptide product formation.^16^ Our results revealed that unmodified Cyclin B1-NT, which is known to be structurally unfolded, was degraded more rapidly by purified 20S proteasome than by 26S proteasome. In contrast, tetra-Ub-Cyclin B1-NT was preferentially degraded by the 26S proteasome. Interestingly, increasing Ub conjugation led to a progressive decrease in degradation by the 20S proteasome, while enhancing degradation by the 26S proteasome. Unexpectedly, we also found that the 20S proteasome is capable of degrading not only the substrate but also the attached ubiquitin tag. These findings challenge the traditional view that the 20S proteasome primarily targets fully unfolded or intrinsically disordered proteins, suggesting instead that it may have a broader role in regulating the proteome. Nevertheless, it remains unclear how the 20S proteasome processes structured proteins bearing polyubiquitin chains.

**Fig. 2:**
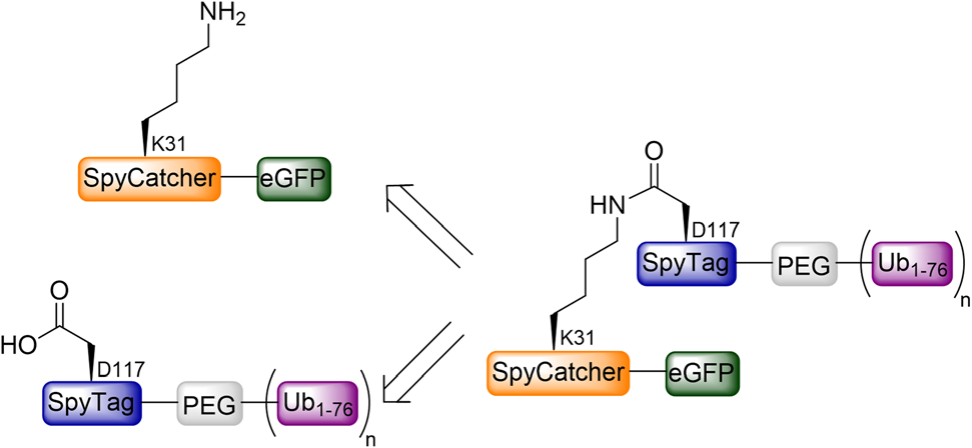
Retrosynthesis for the preparation of eGFP carrying 1–4 Ub units by using the SpyTag/SpyCatcher system.

To test this notation, we generated a panel of homogeneously ubiquitinated eGFP variants as a model for evaluating the SpyTag/SpyCatcher system in protein ubiquitination. eGFP was chosen because it is a folded protein that has been tested for proteasomal degradation to verify various hypotheses regarding 26S proteasomal degradation,^32,33^ which can serve as a valid model for assessing its degradation behavior with the 20S proteasome. To further challenge our system, we opted to link the proximal Ub through the C-terminal of SpyTag/SpyCatcher to eGFP instead of the N-terminal,^34–36^ ensuring that this Ub remains non-cleavable from the substrate.^37^ Therefore, we planned to express the eGFP as a SpyCatcher fusion construct. Simultaneously, the proximal Ub, featuring the SpyTag at its N-terminal, will be prepared as mono-, di-, tri, and tetra-Ub and subsequently linked to the eGFP through the known rapid SpyTag/SpyCatcher reaction. Within this reaction, an isopeptide bond is formed between the lysine side chain of the SpyCatcher protein (113 AA) and the aspartate side chain of the SpyTag peptide (16 AA) (Fig. 2).^38^ The SpyTag/SpyCatcher reaction has been utilized for various applications,^39^ including immobilization of branched SpyTag-Ub chains on SpyCatcher agarose beads for mechanistic studies,^40^ but it has not been applied to protein ubiquitination.

**Fig. 3:**
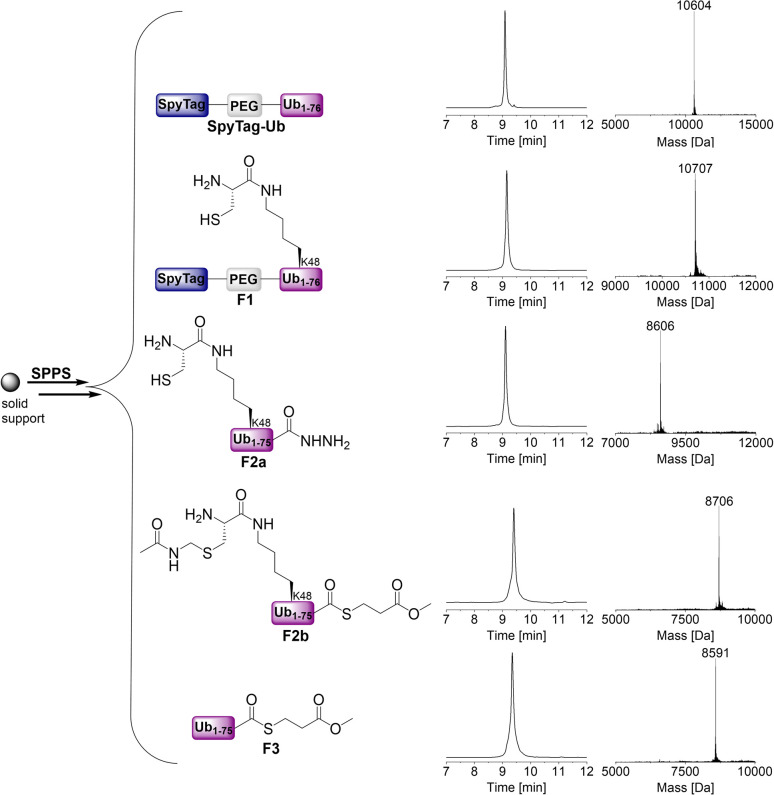
Synthesis of SpyTag-Ub and the required building blocks for the preparation of the SpyTag-Ub variants using SPPS. Calculated masses: SpyTag-Ub: 10 606 Da, F1: 10 709 Da, F2a: 8606 Da, F2b: 8706 Da, F3: 8591 Da.

In the first step, we prepared the SpyTag, which carries one to four Ub chains, using chemical protein synthesis. For the monoubiquitinated SpyTag, we maximized the potential of solid-phase peptide synthesis (SPPS) to nearly 100 amino acids directly on solid support. However, it was necessary to combine SPPS with native chemical ligation (NCL) steps to synthesize longer Ub chains. To synthesize di- to tetra-ubiquitinated SpyTag, we generated four different building blocks (F1, F2a, F2b, and F3) with required functionalities using SPPS (Fig. 3).

**Fig. 4:**
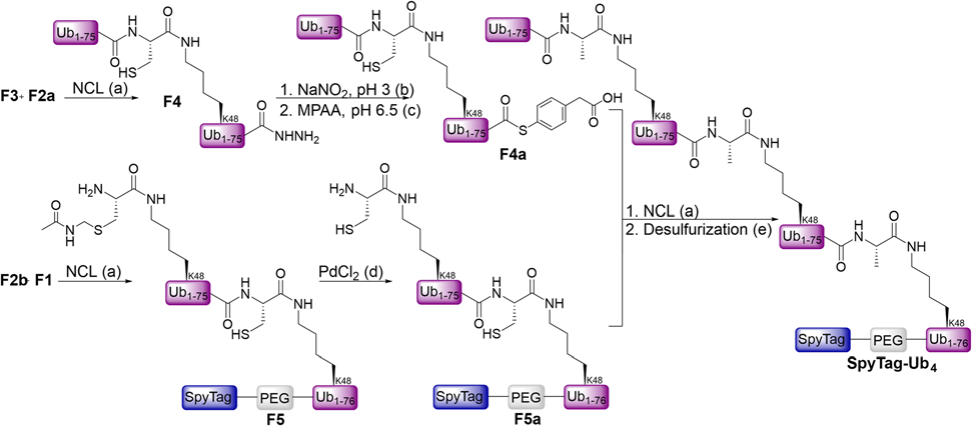
Synthetic scheme for the synthesis of SpyTag-Ub_4_. All reactions were performed in 6 M Gnd-HCl, 200 mM NaPi. (a) 30 eq. TCEP, 50 eq. MPAA, pH 7, 37 °C; (b) 20 eq. NaNO_2_, pH 3, −15 °C; (c) 60 eq. MPAA, pH 6.5, rt; (d) 15 eq. PdCl_2_, 37 °C; (e) 250 mM TCEP, 120 eq./cysteine VA-044, 10% tBuSH, pH 7, 42 °C.

The tetra-Ub chain was prepared through convergent synthesis as described in Fig. 4. The Ub-thioester (F3) was ligated to the Cys-Ub (F2a) to obtain the first di-Ub (F4), while ligation of the Ub-thioester (F2b) with the Cys-SpyTag-Ub (F1) gave the second di-Ub (F5). Following the conversion of the hydrazide moiety in F4 to the 4-Mercaptophenylacetic acid (MPAA) thioester^41^ to give F4a (15% isolated yield over 2 steps) and the deprotection of Acm using PdCl^42^ in F5 to give F5a (14% isolated yield over 2 steps), NCL of the two fragments combined with *in situ* desulfurization^43^ provided access to the SpyTag-Ub_4_ protein. All SpyTag-Ub variants were obtained in high purity, as evident by HPLC and MS, as well as by gel analyses with Coomassie stain (Fig. S1).

**Fig. 5:**
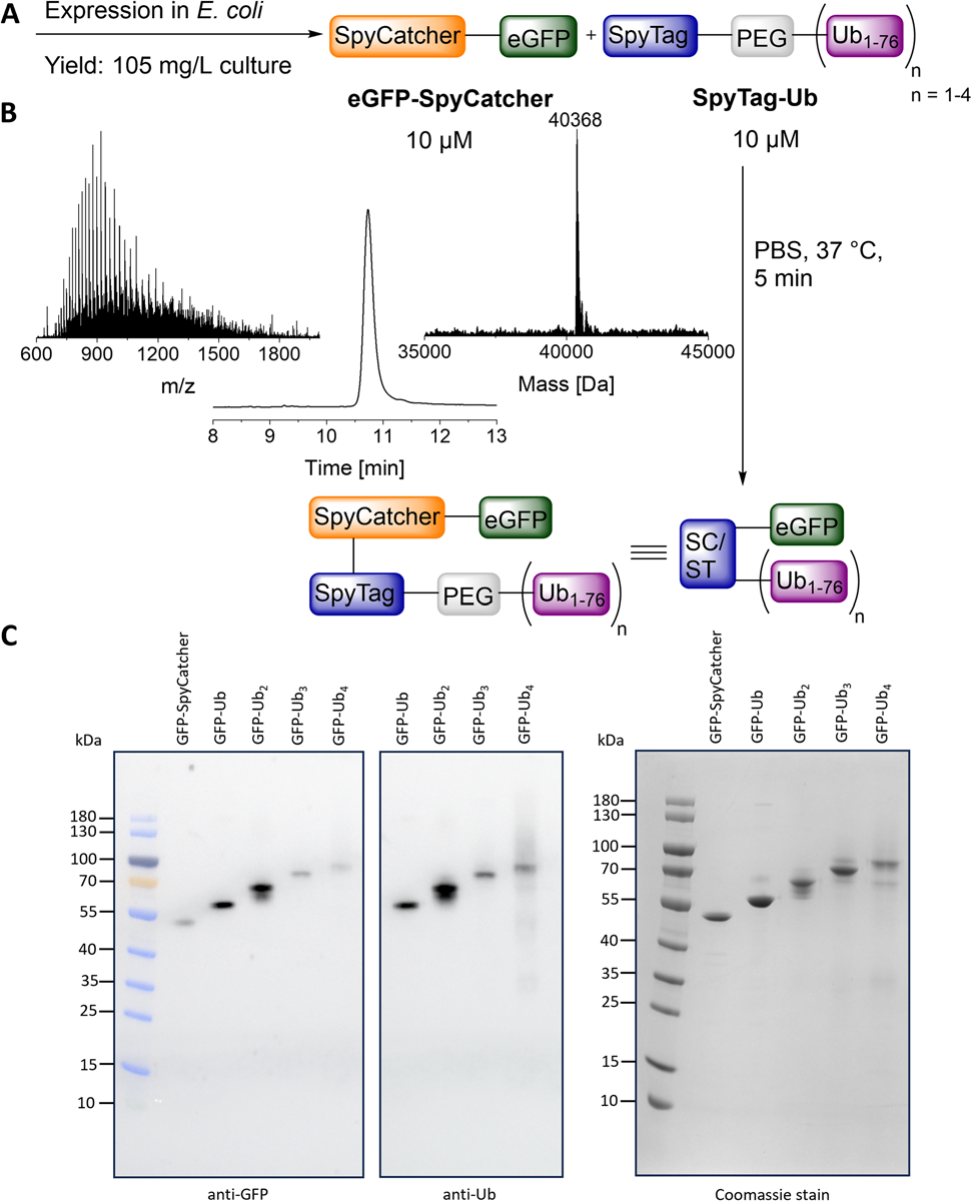
Conjugation of eGFP-SpyCatcher with the SpyTag-Ub variants. (A) General reaction scheme. (B) HPLC-MS analysis of purified eGFP-SpyCatcher. (C) Gel analysis of purified eGFP-SpyCatcher and purified eGFP-Ub conjugates. Molecular weights: eGFP-SpyCatcher: 40 365 Da, eGFP-Ub: 50 953 Da, eGFP-Ub_2_: 59 497 Da, eGFP-Ub_3_: 68 036 Da, eGFP-Ub_4_: 76 577 Da.

Our target protein (eGFP) was expressed in *E. coli* as a fusion construct with SpyCatcher,^31^ which is necessary for conjugation to the SpyTag-carrying Ub chains (Fig. 5A). The fusion construct was obtained in high yield (105 mg per L culture) and purity (Fig. 5B). For the conjugation reaction, 10 μM eGFP-SpyCatcher and 10 μM SpyTag-Ub were incubated in PBS buffer (pH 7.4) at 37 °C, and the isopeptide bond between the SpyTag and SpyCatcher was quickly formed within 5 minutes. This allowed us to prepare ubiquitinated eGFP variants with molecular weights up to 76 kDa. All conjugates were obtained in high purity, as confirmed by western blotting against anti-GFP and anti-Ub, as well as Coomassie staining (Fig. 5C). For the eGFP conjugates carrying one to four Ub units we obtained yields of 14, 8, 3 and 2%, respectively.

**Fig. 6:**
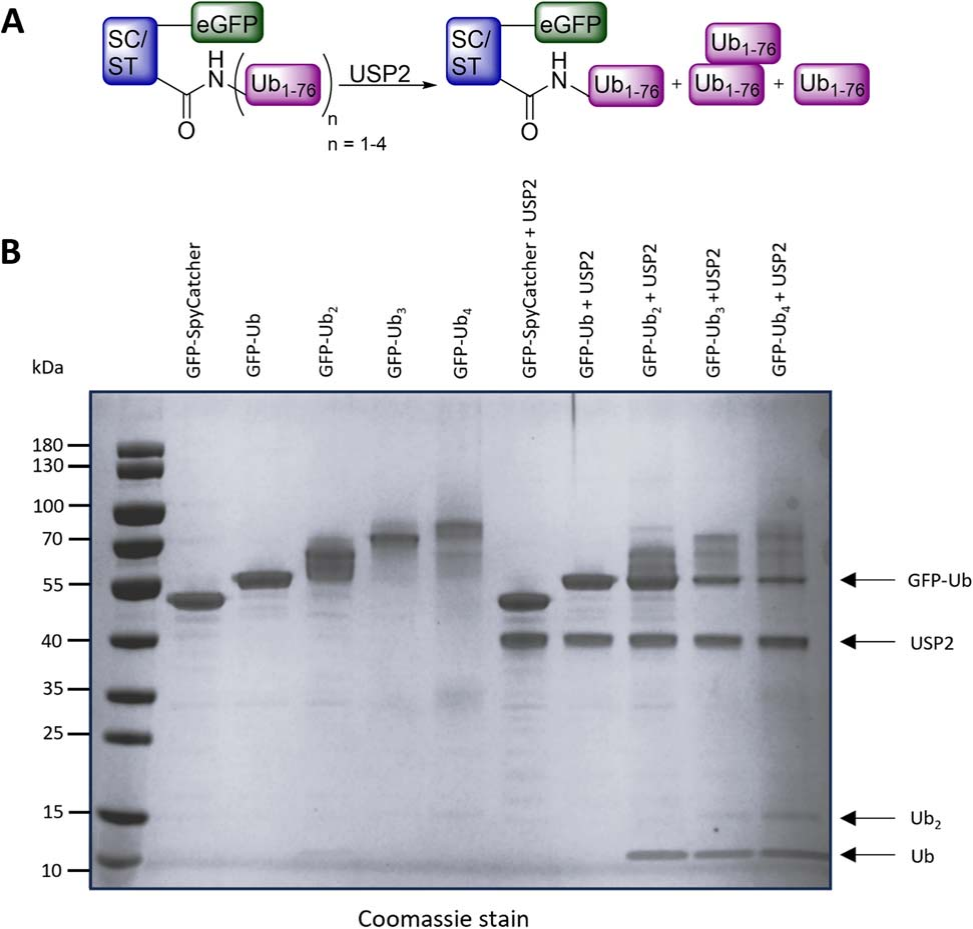
Deubiquitination of the eGFP-SpyCatcher-Ub conjugates by USP2. (A) General reaction scheme. (B) Gel analysis of eGFP-SpyCatcher-Ub conjugates before deubiquitination and after incubation with USP2 for 2 h.

With the four conjugates available, we examined their deubiquitination in the presence of USP2. As expected, monoubiquitinated eGFP was not recognized as a USP2 substrate due to the orientation of the Ub. However, the eGFP variants containing two to four Ub molecules were successfully deubiquitinated, as demonstrated by gel analysis using Coomassie stain (Fig. 6), confirming that the eGFP-Ub conjugates were also properly folded. For eGFP-di-Ub, the release of mono-Ub was observable, while for tri- and tetra-ubiquitinated eGFP, free di-Ub was also evident.

**Fig. 7:**
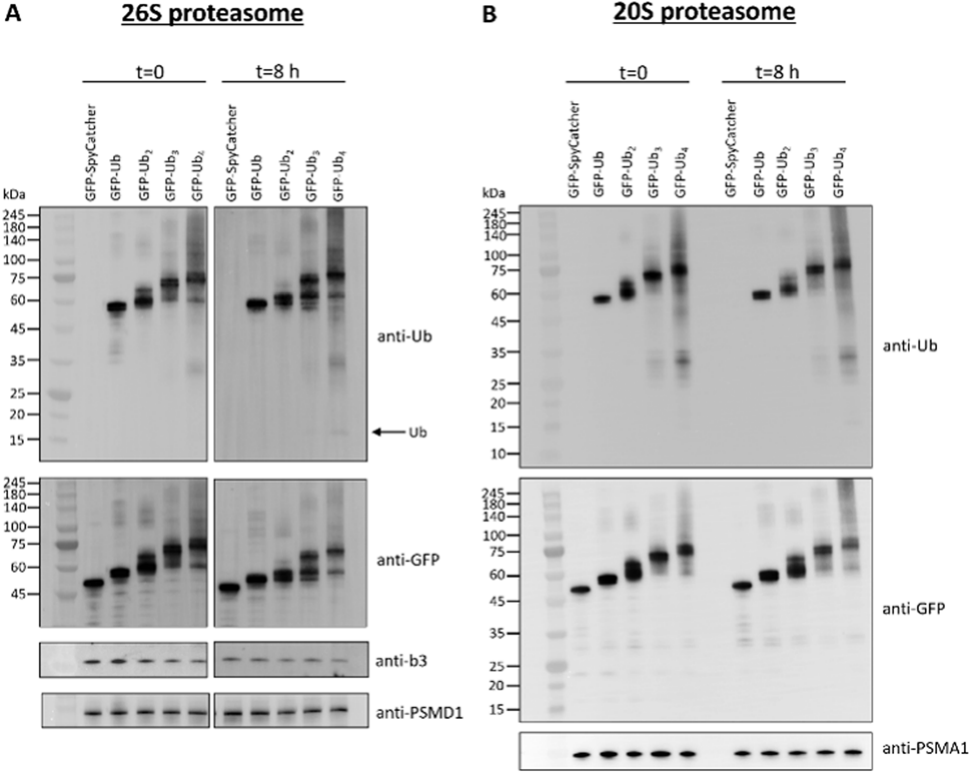
Proteasomal degradation of eGFP-Ub conjugates. (A) Degradation by the 26S proteasome. (B) Degradation by the 20S proteasome. Antibodies against PSMD1 (19S proteasome non-ATPase regulatory subunit 1), PSMA1 and b3 (20S proteasome subunit alpha type-1 and beta type-3 subunit, respectively) were used as proteasome loading controls.

Next, we compared the degradation of our eGFP-Ub conjugates by the 26S and 20S proteasome. When testing these conjugates with the 26S proteasome, only the Ub chains were trimmed by the proteasome-associated DUBs USP14 and USP15 present in our purified 26S proteasome without significant degradation of the substrate (Fig. 7A), probably due to the linkage type with the first Ub. After binding a ubiquitinated protein, the 26S proteasome disassembles and releases the Ub chains by the associated DUBs.^44^ The metalloprotease Rpn11, also present in our purified 26S proteasome, plays a crucial role in removing the Ub chain close to the substrate backbone.^45^ Due to the unnatural connectivity of the first Ub to the substrate, this Ub unit cannot be removed, resulting in trimming of the Ub chain, but not degradation. Furthermore, it has been demonstrated that incomplete degradation and release of globular proteins, which are difficult to unfold (*e.g.*, GFP), often occur with the purified 26S proteasome.^46^ An unstructured region is required in the protein to serve as an initiation site for degradation,^47^ which is likely missing in our Ub conjugates.

On the other hand, the 20S proteasome exhibited apparent degradation of the conjugates, regardless of the connectivity of the first Ub (Fig. 7B). In contrast to the 26S proteasome, no Ub was released; instead, it was degraded completely along with the substrate. Although the 20S proteasome is known to degrade proteins through a Ub-independent pathway,^48^ our results indicate an affinity of the 20S proteasome for tetra-Ub. The longer the Ub chain is, the more protein gets degraded (after 8 h: Ub: 8%, Ub_2_: 31%, Ub_3_: 39%, Ub_4_: 65%, see Fig. S2), which clearly shows that the Ub has an influence on degradation efficiency by the 20S proteasome.

## Conclusions

We presented a novel strategy for preparing ubiquitinated proteins by leveraging chemical protein synthesis, protein expression, and the unique reactivity of the SpyTag/SpyCatcher system. Using this method, we generated a panel of ubiquitinated eGFP with unique connectivity of the proximal Ub to the substrate and utilized it to investigate degradation by the 26S and 20S proteasomes. While the 26S proteasome only trimmed the Ub chain due to the connectivity between the substrate and the proximal Ub, which DUBs cannot recognize, the 20S proteasome degraded the Ub conjugates nonspecifically. Interestingly, our results indicate an affinity of the 20S proteasome for tetra-Ub, although it is known to degrade proteins independently of Ub. These findings raise questions about how Ub influences degradation by the 20S proteasome and whether longer or branched Ub chains might lead to even faster degradation. Since our established ubiquitination method is highly flexible, it can be further utilized to generate eGFP-Ub conjugates with a wide variety of Ub chains to study their influence on degradation in detail. The obvious advantages of using the SpyTag/SpyCatcher system for protein ubiquitination are the high specificity, rate and efficiency of the reaction without any additives. In addition, it is an easy-to-handle reaction and it is performed under native conditions to maintain folding of the target protein.

While ubiquitinated eGFP is a useful system for studying protein degradation, it is important to highlight that our method is not limited to eGFP. In principle, any protein of interest that can be expressed as a SpyCatcher fusion protein can be ubiquitinated using our new approach. Importantly, the SpyTag/SpyCatcher reaction is compatible with different buffer and pH conditions, which can then be adjusted based on the protein of interest. This broadens the toolkit for creating poly-ubiquitinated proteins and will support further research on ubiquitination across various proteins.

## Author contributions

J. K. prepared the SpyTag-Ub variants and the eGFP-Ub conjugates. S. L. performed the proteasomal degradation experiments. M. H. expressed eGFP and conducted the deubiquitination experiment. E. N. assisted with peptide synthesis. J. K. and A. B. wrote the original draft, and S. L., M. H., and M. G. reviewed and edited the manuscript.

## Conflicts of interest

There are no conflicts of interest to declare.

## Supplementary Material

SC-016-D5SC05440K-s001

## Data Availability

The data supporting this article have been included as part of the SI. Supplementary information: Peptide synthesis, conjugation reactions, deubiquitination and degradation assays. See DOI: https://doi.org/10.1039/d5sc05440k.
