# Supplementary material for: SpyTag/SpyCatcher-mediated protein ubiquitination to investigate 20S and 26S proteasomal degradation
Source: Chem Sci. 2025 Sep 10;16(40):18673–8. doi: 10.1039/d5sc05440k (PMC12434616; doi:10.1039/d5sc05440k)
Supplement: SC-016-D5SC05440K-s001 [file SC-016-D5SC05440K-s001.pdf]

Supplementary Information (SI) for Chemical Science.
This journal is © The Royal Society of Chemistry 2025

Supporting Information

SpyTag/SpyCatcher-mediated protein ubiquitination to investigate 20S and 26S proteasomal degradation

Julia Kriegesmann[1], Shahar Levi[2], Mahdi Hasan[1], Eman Nassar[1], Michael Glickman[2] and Ashraf Brik[1]

[1]Schulich Faculty of Chemistry, Technion – Israel Institute of Technology, Haifa, Israel

[2]Faculty of Biology, Technion – Israel Institute of Technology, Haifa, Israel

Correspondence to: abrik@technion.ac.il

Table of Contents

Supplementary Figures 3
General Information..4
Peptide Synthesis, Purification and Analysis 4
Synthesis of the Different mono-Ub Fragments..5
Synthesis of F1..5
Synthesis of F2a..6
Synthesis of F2b..7
Synthesis of F3..9
Synthesis of the Different di-Ub Fragments..9
Synthesis of F4a..10
Synthesis of F5a..10
Synthesis of the Different Ub Variants..12
Synthesis of SpyTag-Ub 12
Synthesis of SpyTag-Ub_2..12
Synthesis of SpyTag-Ub_3..14
Synthesis of SpyTag-Ub_4..15
SpyCatcher-eGFP Expression and Purification..15
Conjugation of the Ubiquitin Variants with the SpyCatcher-eGFP Protein..15
Purification and Analysis of the Ub-SpyTag-SpyCatcher-eGFP Conjugates 16
Gel Analysis and Western Blot..16
Deubiquitination of the Conjugates by USP2..17
Proteasomal Degradation of the Conjugates..17
Native gel, in-gel activity assay, and native immunoblotting..18
References..20

Supplementary Figures

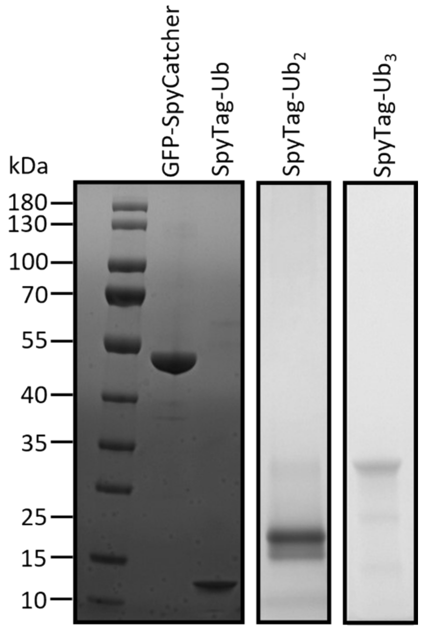

Figure S1: Gel analysis of SpyCatcher-eGFP and three SpyTag-Ub variants with Coomassie stain. For **SpyTag-Ub$_4$**, the obtained amount was too small to see a clear band.

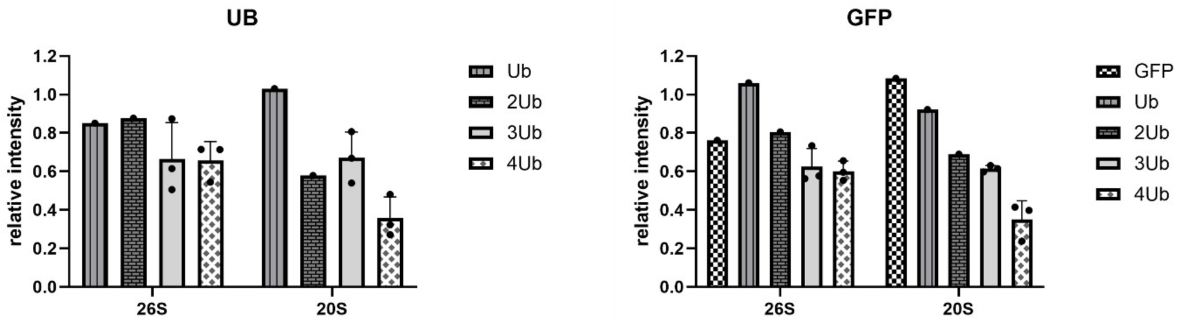

Figure S2: Comparison of proteasomal degradation of the eGFP-Ub variants by the 26S and 20S proteasome. Band intensities detected with GFP or ubiquitin antibodies for all the different conjugates were quantified from Western blots at 0 h and after 8 h incubation with purified 26S or 20S proteasomes using LI-COR Image Studio Software (version 6.1.0.79, RRID:SCR_015795). For each conjugate, degradation was calculated as the ratio of band intensity at 8 h relative to its corresponding 0 h value (set as 1). Data represent mean ± SD from n = 3 independent experiments.

General Information

All commercially available chemicals and solvents were purchased from BioLab, J. T. Baker or Sigma Aldrich (Merck) and used without further purification. Solvents for peptide synthesis and chromatography were of "peptide synthesis grade" and "HPLC grade", respectively. Resin, protected Fmoc- and Boc-amino acids and coupling reagents were purchased from Chem-Impex Int'l inc., GL Biochem, Creosalus and Luxembourg Bio Technologies. The medium and buffers for protein expression and purification were prepared with substances from Sigma Aldrich (Merck), Thermo Scientific and Fisher Bioreagents.

Peptide Synthesis, Purification and Analysis

The ubiquitin (Ub) fragments were synthesized on rink amide MBHA resin on a synthesizer (CSBio CS336X automated peptide synthesizer). After swelling in DMF for 1 h, Fmoc deprotection was performed by addition of 20% piperidine in DMF for 2 x 5 min. The resin was washed 3 x with DMF and the amino acid (AA) was coupled for 45 min (4 eq. AA, 4 eq. HCTU, 8 eq. DIPEA). In case double couplings were performed, the coupling time was reduced to 2 x 30 min. For dipeptides, 2.5 eq. AA, 2.5 eq. HATU and 5 eq. DIPEA were used during the coupling step. Underlined AA were coupled as dipeptides and for bold AA double couplings were performed (see chapter "Synthesis of the Different Ub Fragments" for the synthesized sequences). The last amino acid in the sequence was used in its Boc-protected form. The peptide was cleaved from the resin by incubation in cleavage solution (95% TFA, 2.5% H_2O, 2.5 % TIPS) for 3 h. Afterwards, the peptide was precipitated with cold diethyl ether. After centrifugation (4000 rpm, 10 min, 4°C), the pellet was dissolved in 50% ACN in H_2O and lyophilized.

The lyophilized peptide was dissolved in 6 M Gnd-HCl pH 4.7 and purified by preparative high performance liquid chromatography (HPLC) on a Dionex Ultimate 3000 system (Thermo Scientific). H_2O + 0.05% TFA and ACN + 0.05% TFA were used as buffers A and B, respectively. Gradients from 5-65% B in 20-40 min were used with a flow rate of 15 mL/min. The mass of the peptides was confirmed by an LCQ Fleet Ion Trap (Thermo Scientific) and the purity was investigated by analytical HPLC (5-65% B in 30 min, 1.2 mL/min), using the same buffers as for preparative HPLC.

Synthesis of the Different mono-Ub Fragments

Synthesis of F1

RGVPHIVMVDAYKRYK-[PEG]-
NleQIFVKTLTGKTITLEVEPSDTIENVKAKIQDKEGI**P**PDQQRLIFAG**K(Dde)$_{48}$**QL
EDGRTLSDYNIQKESTLHLVLRLRGG$_{76}$

Ub$_{1\text{-}76}$ and the SpyTag, linked by PEG, were synthesized by the general peptide synthesis method. At position K$_{48}$, Fmoc-Lys(Dde)-OH was coupled. After completion of the synthesis, the Dde protection group was removed selectively by incubation with 5% hydrazine solution in DMF for 3 x 30 min and Boc-Cys(Trt)-OH was coupled. The peptide was cleaved and purified following the general procedure.

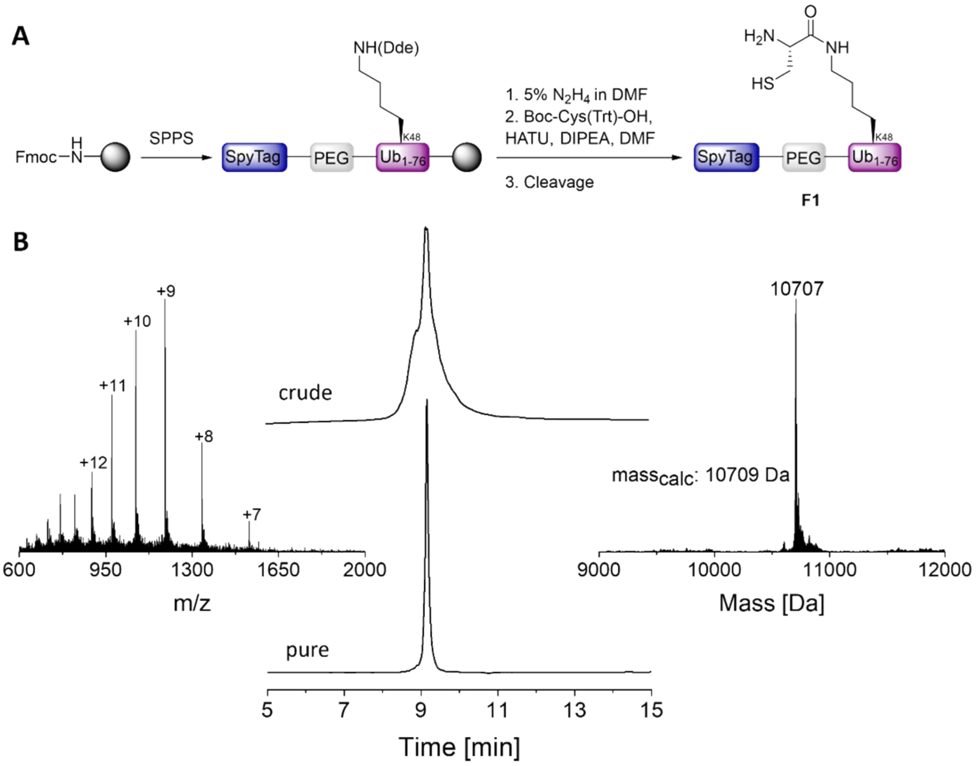

Figure S3: Synthesis of **F1**. A: Synthesis scheme. B: Analysis of the crude and purified peptide.

Synthesis of F2a

NleQIFVKTLTGKTITLEVEPSDTIENVKAKIQDKEGI**P**PDQQRLIFAG**K(Dde)$_{48}$**QLEDGRTLSDYNIQKESTLHLVLRLRG$_{75}$

The hydrazide resin was prepared by using CTC resin. A solution of 10% hydrazine in DMF was added to the resin and incubated for 2 x 30 min. After washing (3 x DMF, 3 x DCM, 3 x DMF), a capping step with 5% MeOH in DMF was performed for 20 min. The resin was washed again (3 x DMF, 3 x DCM, 3 x DMF) and Fmoc-Gly-OH was coupled (1 eq. AA, 0.9 eq. HATU, 2 eq. DIPEA). For capping of the resin, Boc-Gly-OH was coupled (2 eq. AA, 1.9 eq. HATU, 4 eq. DIPEA). A resin loading of 0.22 mmol/g was obtained. The Ub sequence was synthesized by regular SPPS and as a last amino acid Boc-Nle-OH was coupled. The peptide was cleaved and purified following the general procedure.

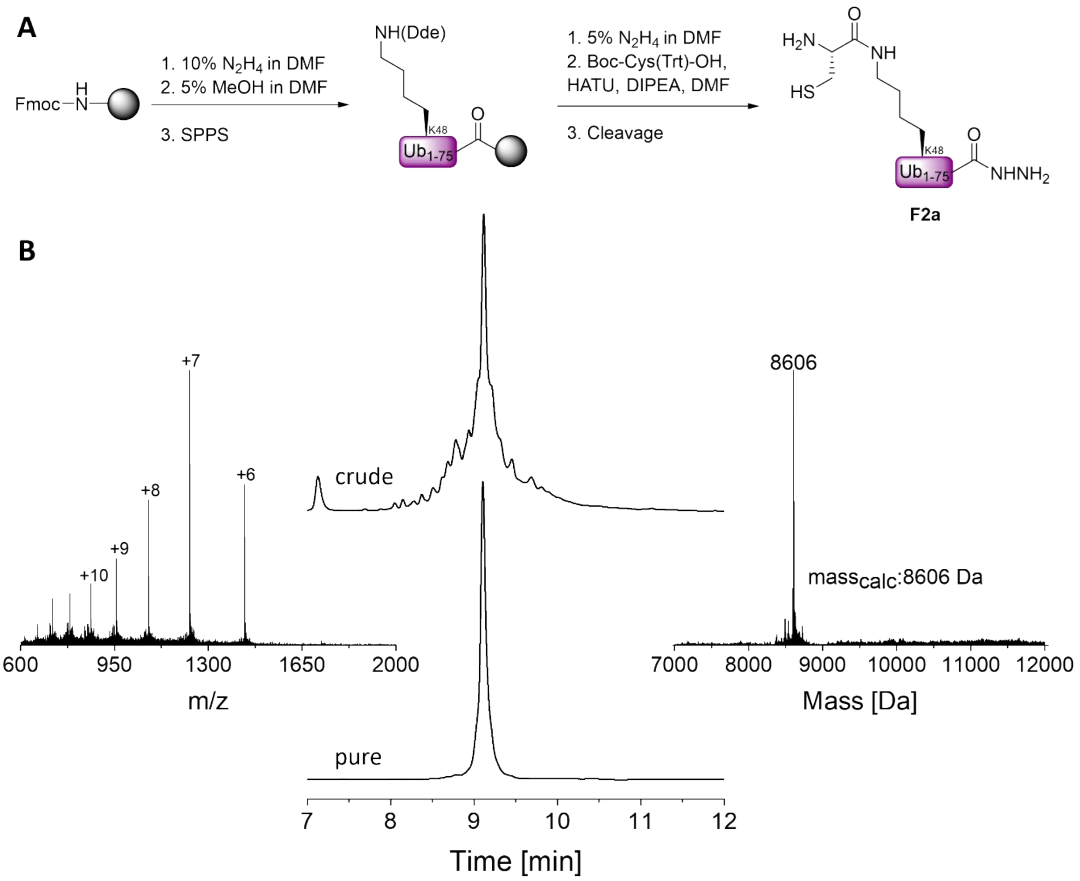

Figure S4: Synthesis of **F2a**. A: Synthesis scheme. B: Analysis of the crude and purified peptide.

Synthesis of F2b

NleQIFVKTLTGKTITLEVEPSDTIENVKAKIQDKEGI**P**PDQQRLIFAG**K(Dde)$_{48}$**QLEDGRTLSDYNIQKESTLHLVLRLRG$_{75}$

Fmoc-DBZ-OH was coupled to the resin, followed by Alloc protection of the free amine. Therefore, the resin was washed 3 x with DMF and DCM and a solution of 1060 µL allyl chloroformate and 140 µL DIPEA in DMF was added (for 0.2 mmol scale). After incubation for 16 h under exclusion of light, the resin was washed with 3 x DCM and 3 x DMF. The following Ub sequence was synthesized by regular SPPS. At position K_{48}, Fmoc-Lys(Dde)-OH was coupled. After completion of the synthesis, the Dde protection group was removed selectively by incubation with 5% hydrazine solution in DMF for 3 x 30 min and Boc-Cys(Acm)-OH was coupled. After washing (3 x DMF and 3 x DCM), the Alloc group was removed by incubating the resin in a solution containing 20 eq. $PhSiH_3$ and 0.2 eq. $Pd(PPh_3)_4$ in DMF for 1.5 h. The resin was washed 3 x with DCM. For cyclization, first a solution of 5 eq. *p*-nitrophenyl chloroformate in 4 mL DCM was added to the resin 3 x 30 min and afterwards it was washed (3 x DCM and 3 x DMF). In a second step, a solution of 10% DIPEA in DMF was added for 3 x 10 min and the resin was washed (3 x DMF and 3 x DCM). The peptide was cleaved and lyophilized following the general procedure. 50 mg of the lyophilized peptides were dissolved in 800 µL of 6 M Gnd-HCl in 200 mM NaP_i, pH 7.2. After addition of 200 eq. MMP, the solution was incubated at 37°C for 1 h and directly purified afterwards using the general purification method.

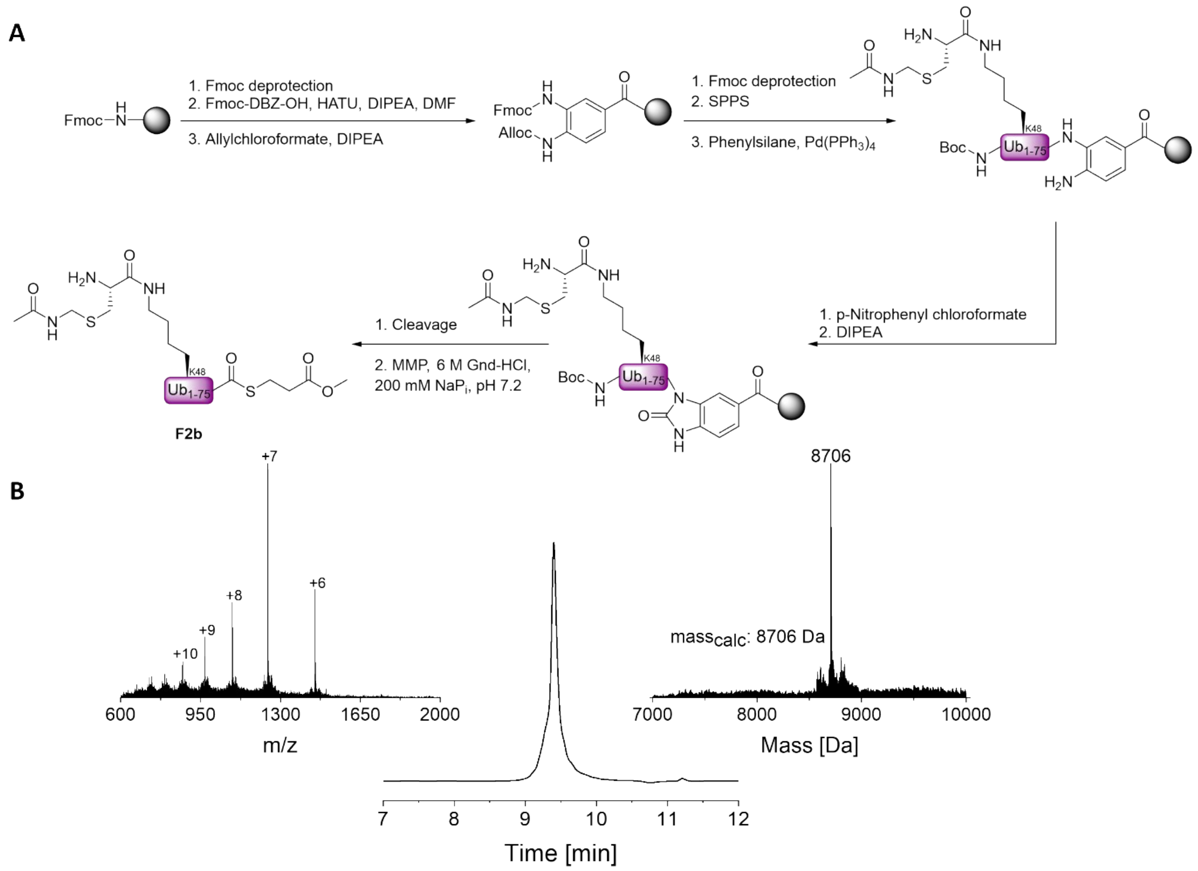

Figure S5: Synthesis of **F2b**. A: Synthesis scheme. B: Analysis of the purified peptide.

Synthesis of F3

All synthesis steps for the preparation of **F3** were carried out exactly as for **F2b**, but at position K_{48}, a regular lysine was coupled.

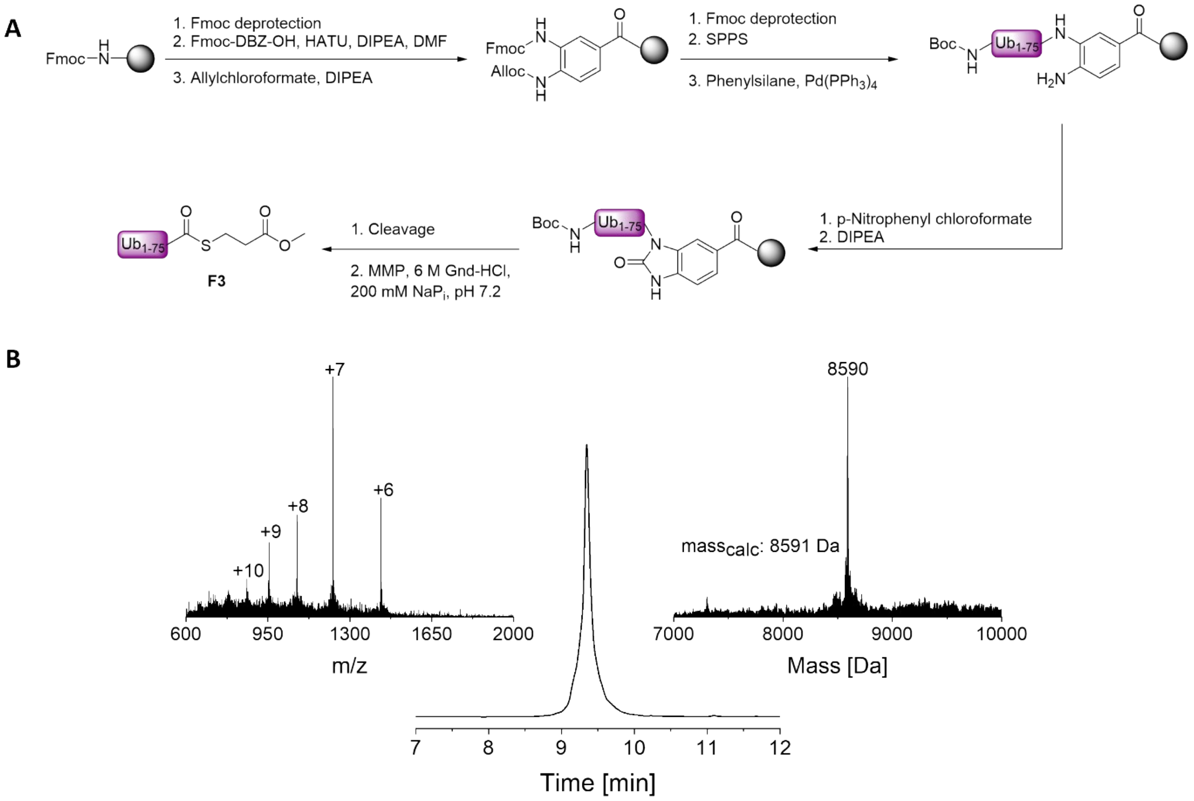

Figure S6: Synthesis of **F3**. A: Synthesis scheme. B: Analysis of the purified peptide.

Synthesis of the Different di-Ub Fragments

The different di-Ub fragments were prepared by native chemical ligation of two mono-Ub fragments. Therefore, 1 eq. of the cysteine-peptide and 1.5 eq. of the thioester peptide were dissolved in ligation buffer (6 M Gnd-HCl, 200 mM NaP_i, 30 eq. TCEP, 50 eq. MPAA, pH 7) to obtain a final concentration of 2 mM for the cysteine-peptide. The reactions were carried out at 37°C and monitored by analytical HPLC. After full conversion to the desired di-Ub was achieved, the reaction mixture was dialyzed against 6 M Gnd-HCl in 200 mM NaP_i, pH 7.2 at rt for 16 h.

Synthesis of F4a

The fragments **F2a** and **F3** were ligated based on the general ligation procedure, followed by dialysis against 6 M Gnd-HCl, 200 mM NaP_i, pH 7 and purification by size exclusion chromatography (SEC) on an ÄKTA Avant System with a Superdex™ 75 Increase 10/300 GL column. 6 M Gnd-HCl, 200 mM NaP_i, pH 7.2 was used as solvent. Fractions were checked for purity by gel analysis with Coomassie staining as described below. For the conversion of **F4** from hydrazide to MPAA-ester, the pH was adjusted to 3.1 and the peptide was incubated at -15°C for 15 min. 20 eq. $NaNO_2$ were dissolved in H_2O and cooled to 0°C for 15 min. This solution was added to the peptide solution and incubated at -15°C for 15 min. 60 eq. MPAA were dissolved in 6 M Gnd-HCl, 200 mM NaP_i and the pH was adjusted to 6.5-6.7. This solution was added to the peptide solution and incubated at rt for 1 h. Afterwards, the peptide **F4a** was purified by semipreparative HPLC.

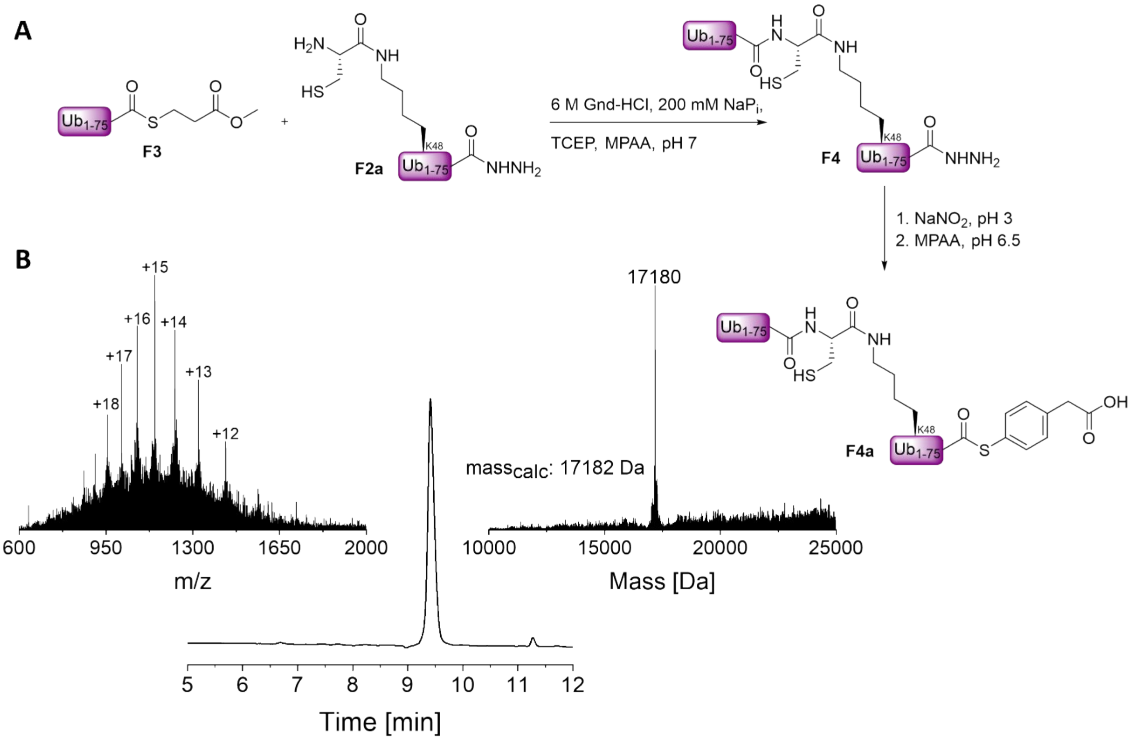

Figure S7: Synthesis of **F4a**. A: Synthesis scheme. B: Analysis of the purified peptide.

Synthesis of F5a

The fragments **F1** and **F2b** were ligated based on the general ligation procedure, followed by dialysis against 6 M Gnd-HCl, 200 mM NaP_i, pH 7 and purification by SEC. 6 M Gnd-HCl, 200 mM NaP_i, pH 7 was used as solvent. Fractions were checked for purity by gel analysis

with Coomassie staining as described below. For the Acm removal of **F5**, 15 eq. $PdCl_2$ were added to the peptide solution and incubated at 37°C for 1 h. Afterwards, the peptide **F5a** was purified by semipreparative HPLC.

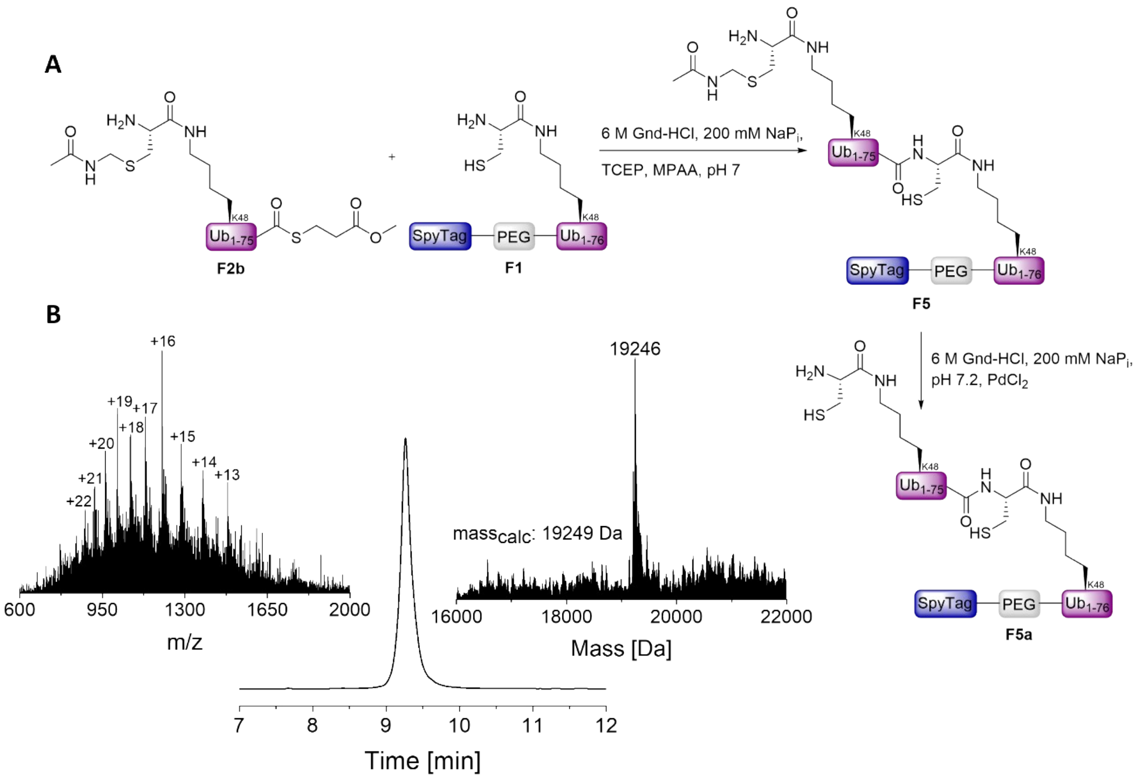

Figure S8: Synthesis of **F5a**. A: Synthesis scheme. B: Analysis of the purified peptide.

Synthesis of the Different Ub Variants

Synthesis of SpyTag-Ub

The synthesis of **SpyTag-Ub** was carried out exactly as for **F1**, but at position K_{48}, a regular lysine was coupled.

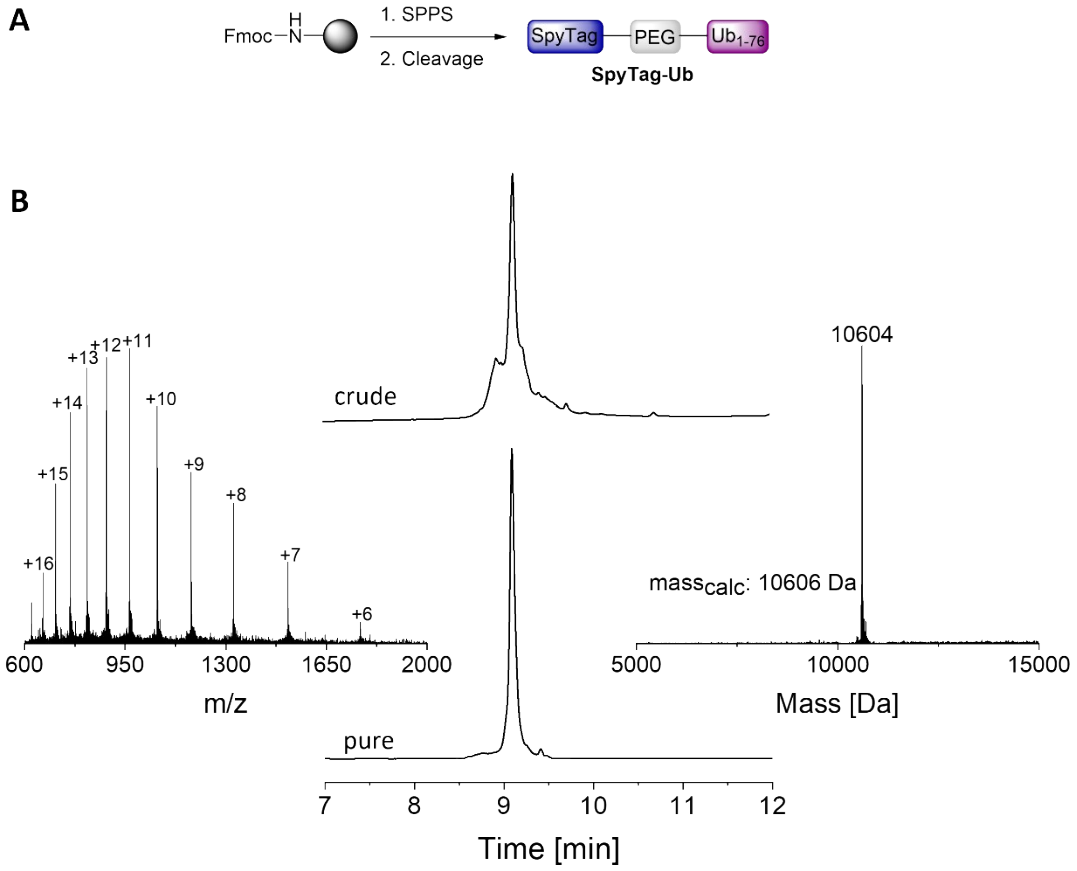

Figure S9: Synthesis of **SpyTag-Ub**. A: Synthesis scheme. B: Analysis of the crude and purified peptide.

Synthesis of SpyTag-Ub$_2$

The fragments **F1** and **F3** were ligated based on the general ligation procedure, followed by dialysis against 6 M Gnd-HCl, 200 mM NaP$_i$, pH 7 and desulfurization. Desulfurization was performed by incubation with a solution of 250 mM TCEP, 120 eq./cysteine VA-044 and 10% *t*BuSH at 42°C for 16 h. The solution was dialyzed against 6 M Gnd-HCl, 200 mM NaP$_i$, pH 7 and purified by SEC. 6 M Gnd-HCl, 200 mM NaP$_i$, pH 7 was used as solvent. Fractions were checked for purity by gel analysis with Coomassie staining as described below. Fractions containing the pure product were dialyzed against PBS.

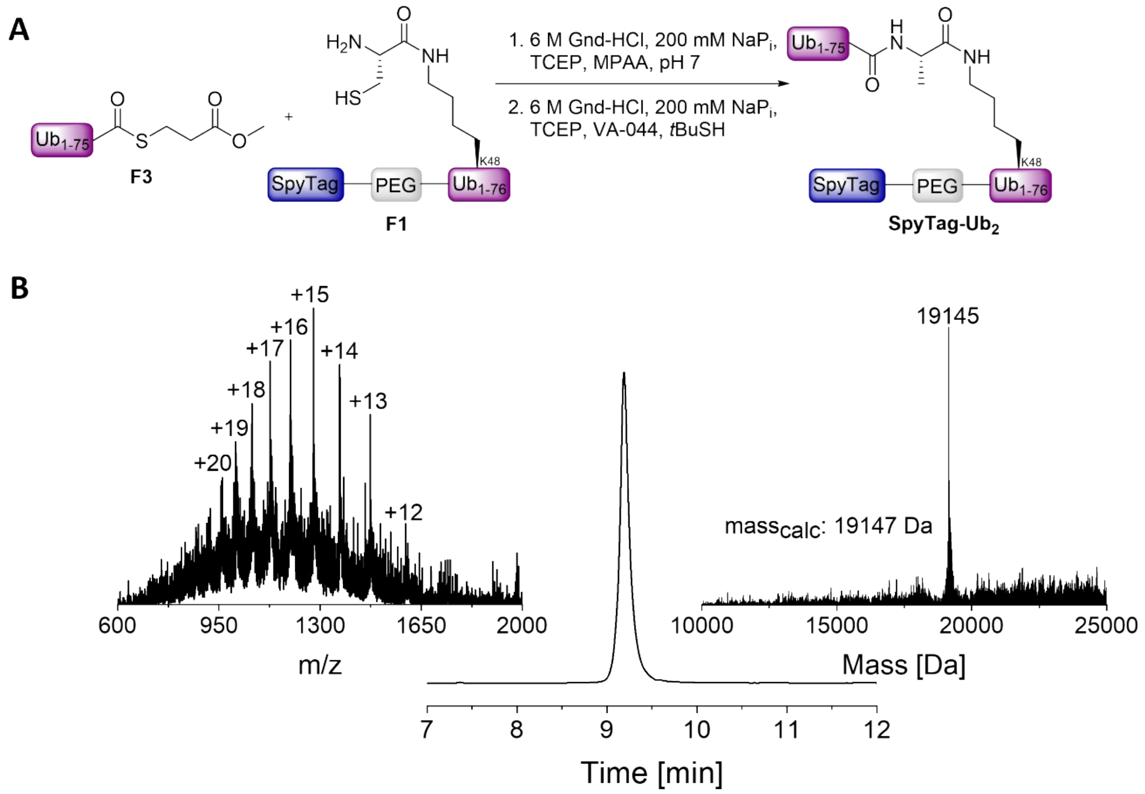

Figure S10: Synthesis of **SpyTag-Ub$_2$**. A: Synthesis scheme. B: Analysis of the purified peptide.

Synthesis of SpyTag-Ub_3

The fragments **F1** and **F4a** were ligated based on the general ligation procedure for 6 h, followed by dialysis, desulfurization and SEC purification as described for **SpyTag-Ub_2**.

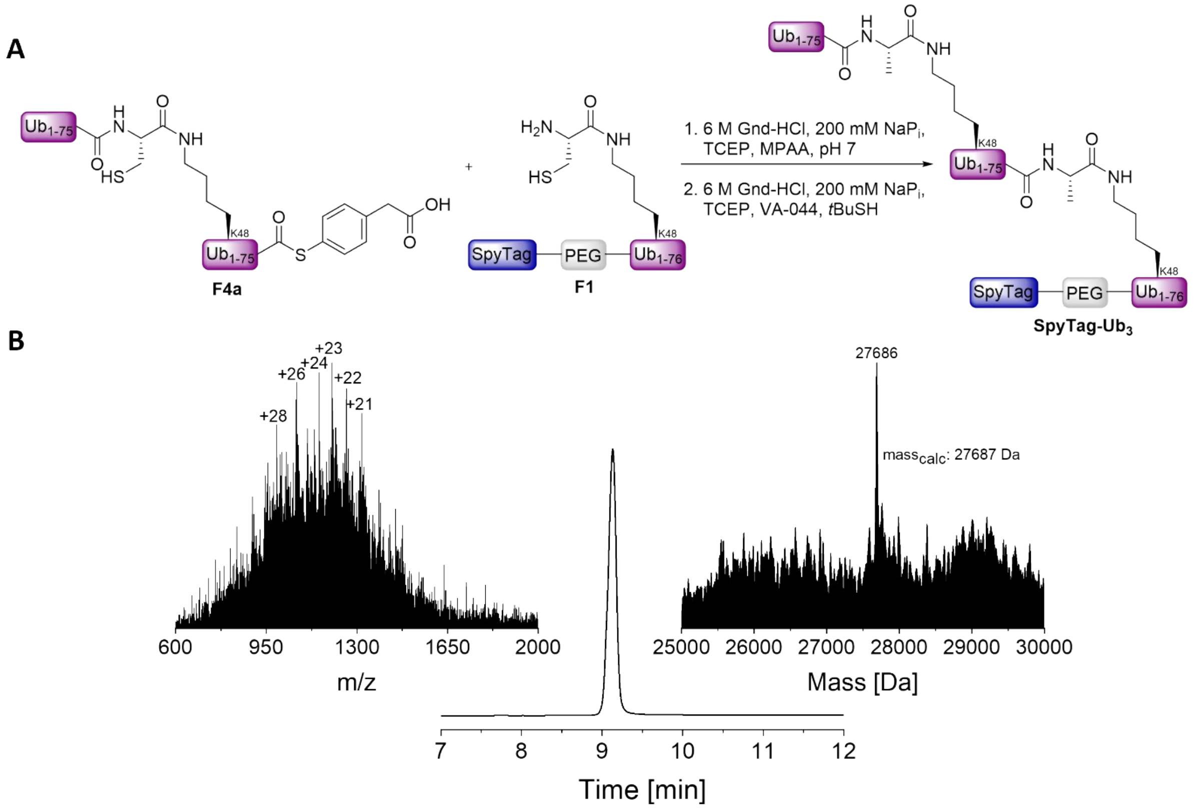

Figure S11: Synthesis of **SpyTag-Ub_3**. A: Synthesis scheme. B: Analysis of the purified peptide.

Synthesis of SpyTag-Ub$_4$

The fragments **F4a** and **F5a** were ligated based on the general ligation procedure for 6 h, followed by dialysis, desulfurization and SEC purification as described for **SpyTag-Ub$_2$**.

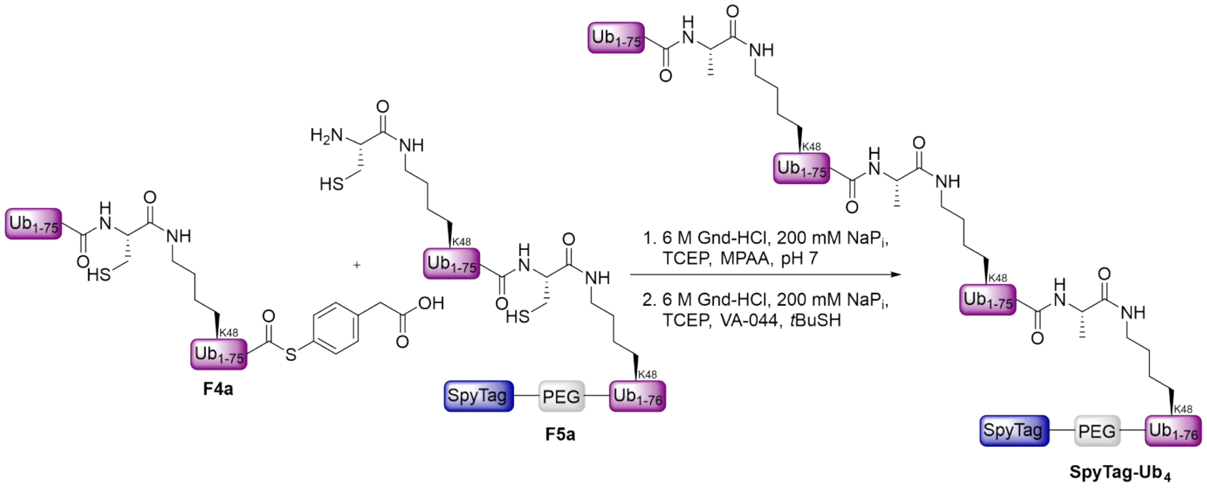

Figure S12: Synthesis scheme for the synthesis of **SpyTag-Ub$_4$**.

SpyCatcher-eGFP Expression and Purification

The SpyCatcher-eGFP was recombinantly expressed in *E. coli* and purified by affinity chromatography based on our established protocol.[1]

Conjugation of the Ubiquitin Variants with the SpyCatcher-eGFP Protein

The SpyCatcher-eGFP (19.2 mg/mL, 475 µM) was diluted with PBS and the respective SpyTag-Ub variant was added to achieve a final concentration of 1 µM for both reactants. The solution was incubated for 5 min at 37°C. Figure S11 shows reaction monitoring by HPLC for conjugate 1. Notably, confirmation of the mass by MS was only possible for the first conjugate. The masses of the other conjugates were confirmed by gel analysis.

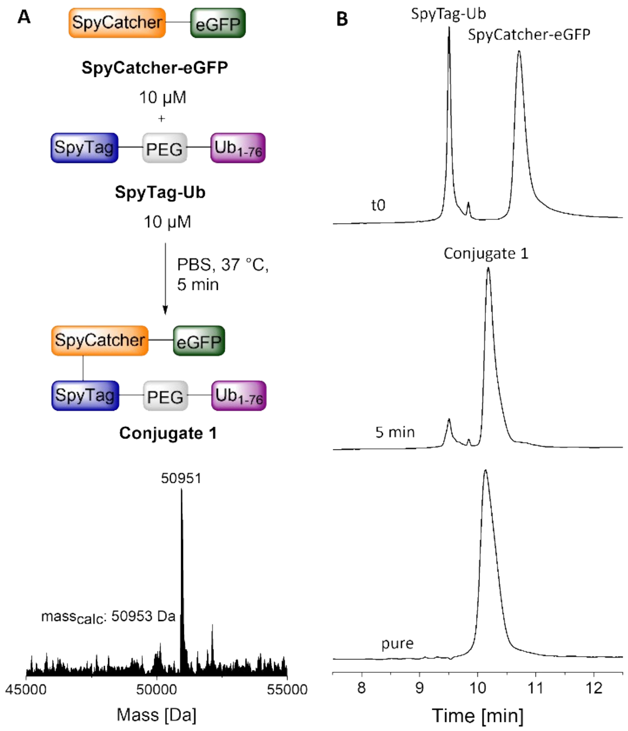

Figure S13: Conjugation reaction of SpyTag-Ub with SpyCatcher-eGFP. A: Reaction scheme. B: Analysis of the reaction at t0, after 5 min and of the product after purification by SEC.

Purification and Analysis of the Ub-SpyTag-SpyCatcher-eGFP Conjugates

The Ub-SpyTag-SpyCatcher-eGFP conjugates were concentrated and purified by SEC. PBS buffer was used as solvent. Fractions were checked for purity by western blot as described below and the fractions containing the pure conjugates were pooled. The conjugates were concentrated and stored at -80°C.

Gel Analysis and Western Blot

For gel analysis, samples were mixed with 6x reducing sample buffer, incubated at 95°C for 5 min and run on a 4-20% MOPS gel at 250 V.

For western blot, the gel was electroblotted onto PVDF membranes followed by blocking with Instant Block Buffer (Gene Bio-Application L.T.D.) for 10 min at rt. After washing, (3 x TBST), the membranes were incubated with anti-Ub or anti-GFP primary antibody (Ub: sc-8017, GFP: SAB4301795) for 16 h at 4 °C overnight. After another washing step (3 x TBST), the membranes were incubated with the corresponding secondary antibodies for 1 h at rt and

intensively washed again (5 x TBST). The membranes were incubated with Immobilon® Crescendo Western HRP substrate (Millipore).

For Coomassie staining, the gel was incubated with staining solution (45% MeOH in H_2O, 10% acetic acid, 0.1% Coomassie R250) for 30 min at rt and destained in H_2O until good contrast was obtained.

All images were taken with a VILBER Fusion FX Imager.

Deubiquitination of the Conjugates by USP2

The 50 µM USP2 stock solution (Bio-Techne) was diluted to a concentration of 2.5 µM with DUB dilution buffer (25 mM Tris, 150 mM NaCl, 10 mM DTT, pH 7.5). For the deubiquitination by USP2, 1 µL 10x DUB buffer (500 mM Tris, 500 mM NaCl, 50 mM DTT, pH 7.5), 0.5 µL 2.5 µM USP2 and the respective conjugate were mixed to obtain a conjugate concentration of 5 µM in 10 µL reaction volume. The deubiquitination was checked by gel analysis with Coomassie staining after 2 h.

Proteasomal Degradation of the Conjugates

26S and 20S proteasome complexes were purified from human erythrocytes as described in our previous work.[2] For each reaction, 220 nM of either 26S or 20S proteasome was mixed with 1 µM of the respective conjugate in a 20 µL reaction. For 26S-mediated degradation, reactions were incubated in buffer A (25 mM Tris pH 7.4, 10 mM $MgCl_2$, 10% glycerol, 1 mM ATP, 1 mM DTT). For 20S-mediated degradation, reactions were incubated in buffer A without ATP and DTT (25 mM Tris pH 7.4, 10 mM $MgCl_2$, 10% glycerol). All reactions were incubated at 37 °C for 0, 8, or 24 h. Samples were then resolved by SDS-PAGE and analyzed by western blotting. From each sample, 100 ng of conjugate (2 µL) was loaded. As a negative control, all conjugates were incubated without proteasome for 8 h. For immunoblotting, proteins were transferred onto a PVDF membrane (0.45 µm, Millipore), followed by blocking with 5% skim milk for 30 min at rt. Membranes were incubated overnight at 4 °C with primary antibodies: anti-PSMD2 (Abcam ab140675), anti-PSMA1 and anti-β3 (from the Hartmann-Petersen lab, University of Copenhagen), anti-**Ub** (Santa Cruz sc-8017), or anti-GFP (Sigma SAB4301795). After washing with TBST, membranes were incubated with the corresponding secondary antibodies for 1 h at rt.

Band intensities detected with GFP or ubiquitin antibodies for all the different conjugates were quantified from Western blots at 0 h and after 8 h incubation with purified 26S or 20S proteasomes using LI-COR Image Studio Software (version 6.1.0.79, RRID:SCR_015795). For each conjugate, degradation was calculated as the ratio of band intensity at 8 h relative to its corresponding 0 h value (set as 1). Data represent mean ± SD from n = 3 independent experiments. Graphs were generated using GraphPad Prism (version 10.6.0 (890) for Windows; GraphPad Software, Boston, Massachusetts USA, www.graphpad.com).

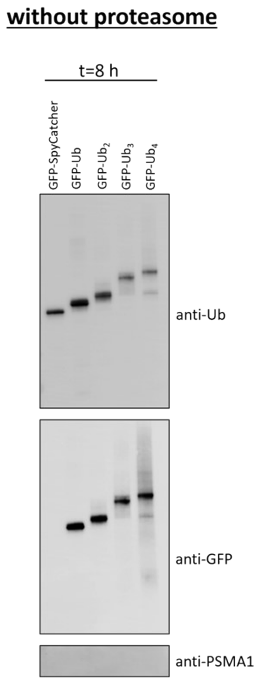

Figure S14: Control reaction for the proteasomal degradation. All conjugates were incubated in buffer A and without proteasome for 8 h.

Native gel, in-gel activity assay, and native immunoblotting

Purified 26S and 20S proteasomes were resolved by 4% native PAGE using ice-cold native running buffer (100 mM Tris base, 100 mM boric acid, 1 mM EDTA, 2.5 mM $MgCl_2$, 0.5 mM ATP, 0.5 mM DTT) for 3 h at 120 V and 4 °C. For in-gel activity assay, the gel was incubated in buffer A containing 25 µM Suc-LLVY-AMC and 0.05% SDS at 37 °C for 15 min and then imaged under UV light. For native immunoblotting, proteins were transferred onto a PVDF membrane (0.45 µm, Millipore), blocked in 5% skim milk for 30 min at rt, and incubated with anti-PSMD2 (Abcam ab140675) overnight at 4 °C. After washing with TBST, membranes were incubated with the corresponding secondary antibodies for 1 h at rt.

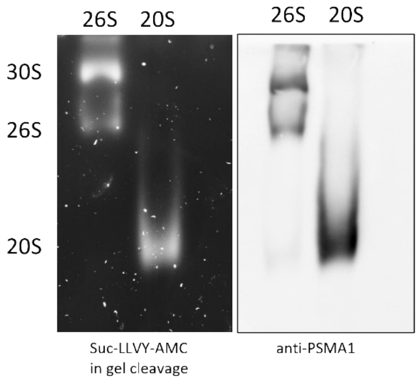

Figure S15: In-gel activity assay of the proteasomes.

References

1 M. Hasan, D. Panda, G. Mann and A. Brik, *ChemBioChem*, 2024, **25**, e202300731.

2 I. Sahu, S. M. Mali, P. Sulkshane, C. Xu, A. Rozenberg, R. Morag, M. P. Sahoo, S. K. Singh, Z. Ding, Y. Wang, S. Day, Y. Cong, O. Kleifeld, A. Brik and M. H. Glickman, *Nat Commun*, 2021, **12**, 6173.
